# The cerebellum shows its stripes

**DOI:** 10.7554/eLife.52631

**Published:** 2019-11-14

**Authors:** Ashley L Holloway, Talia N Lerner

**Affiliations:** 1 Department of PhysiologyFeinberg School of Medicine, Northwestern UniversityChicagoUnited States; 2 Northwestern University Interdepartmental Neuroscience (NUIN) Graduate ProgramEvanstonUnited States

**Keywords:** cerebellum, climbing fiber, reinforcement learning, go/no-go task, Purkinje cells, Mouse

## Abstract

New studies examine how the different sub-structures in the cerebellum are organized to receive information during complex behavioral tasks

**Related research article** Heffley W, Hull C. 2019. Classical conditioning drives learned reward prediction signals in climbing fibers across the lateral cerebellum. *eLife* **8**:e46764. doi: 10.7554/eLife.46764

**Related research article** Tsutsumi S, Hidaka N, Isomura Y, Matsuzaki M, Sakimura K, Kano M, Kitamura K. 2019. Modular organization of cerebellar climbing fiber inputs during goal-directed behavior. *eLife* **8**:e47021. doi: 10.7554/eLife.47021

Tucked away at the back of the brain, the cerebellum is best known for its role in fine-tuning movement and motor activity. However, many believe it is also involved in cognitive functions and that it could contribute to developmental disorders such as autism (Wang et al., 2014). New lines of research have thus begun to tackle how the cerebellum may be involved in motivated behaviors (that is, complex actions directed towards or away from a stimulus; Heffley et al., 2018; Kostadinov et al., 2019; Xiao et al., 2018). Now, in eLife, two studies report how reward-related inputs to the cerebellum are organized. Understanding this organization is a crucial first step to grasp how the structure may help to control motivated behaviors.

The cerebellum receives information from the brain stem through neuronal projections called climbing fibers, which connect to the output neurons of the cerebellar cortex, known as Purkinje cells. While the cerebellum can, at first, look homogeneous, it is in fact arranged into discrete lobules, including Crus I, Crus II and the lobule simplex. In the first study, William Heffley and Court Hull, from Duke University School of Medicine, examined how reward information is received in the lateral portions of these three lobules (Heffley and Hull, 2019).

To do so, they engineered the Purkinje cells of mice so that these cells contained a fluorescent indicator of calcium levels in their dendrites. Calcium increases in Purkinje cell dendrites had been previously shown to be a proxy for electrical activity in the climbing fiber inputs to these cells (Heffley et al., 2018; Tsutsumi et al., 2015). The mice were trained to expect that a specific visual stimulus would be followed by a food reward, and the experiments showed that climbing fibers in the three lobules responded differently during this Pavlovian task.

In the lobule simplex, the responses appeared to track reward predictions. Before the animals had learned to associate the visual stimulus with food, the cells reacted when the reward was delivered. However, these responses faded with learning: instead, they started to appear only in reaction to the cue and before the reward itself. In fact, after training, the responses emerged even if food failed to follow the visual stimulus, as the animal expected a recompense.

The climbing fibers in Crus II reacted in a similar way, but the responses to the reward itself persisted after learning. In contrast, responses in Crus I were related to sensory information: they emerged in reaction to the visual cue, before the food was delivered or the animal had started to consume it.

Within the lobules, the cerebellum is further divided into microzones, each of which receives distinct inputs from climbing fibers and projects to different downstream cerebellar nuclei (Oscarsson, 1979). Moreover, staining these microzones for molecular markers, such as the enzyme Aldolase C (AldC) or the antigen zebrin II, reveals a dramatic striped pattern of Purkinje cells that spans the cerebellar cortex, with alternating AldC+ and AldC- modules within the microzones (Sugihara and Shinoda, 2004; Voogd and Ruigrok, 2004). However, tools that could examine whether these modules have distinct roles were not available. A previous study used dye injections during recordings and post-hoc tissue analysis to categorize Purkinje cells by their expression patterns, but this approach is low-throughput (Zhou et al., 2014).

Therefore, in the second study, Shinichiro Tsutsumi, Masanobu Kano, Kazuo Kitamura and colleagues employed a more efficient approach, using a mouse line where AldC+ Purkinje cells were labeled with a red fluorescent protein (Tsutsumi et al., 2019). This method allowed them to identify AldC+ (red) and AldC- (unlabeled) modules during live calcium imaging experiments. As in the first study, they were then able to use the calcium increases in Purkinje cell dendrites to infer the activity of climbing fiber inputs. In particular, they looked at climbing fiber inputs to AldC+ and AldC- Purkinje cells across the medial and lateral microzones of the Crus II lobule.

The mice in this study were trained on a Go/No-go auditory discrimination task (Figure 1A). A 10 kHz tone predicted the release of a sweet liquid, while a 4 kHz tone was not paired with food. A 10 kHz trial was labeled as a ‘hit’ if the animals responded by licking before the food appeared, and a ‘miss’ if they failed to do so. Licking in response to the 4 kHz tone was a ‘false alarm’, but not licking was a correct rejection. Using this approach, Tsutsumi et al. – who are based at the University of Tokyo, CREST, the University of Yamanashi, Tamagawa University and Niigata University – observed that climbing fiber information about goal-directed behaviors is distributed heterogeneously across Crus II.

**Figure 1.**
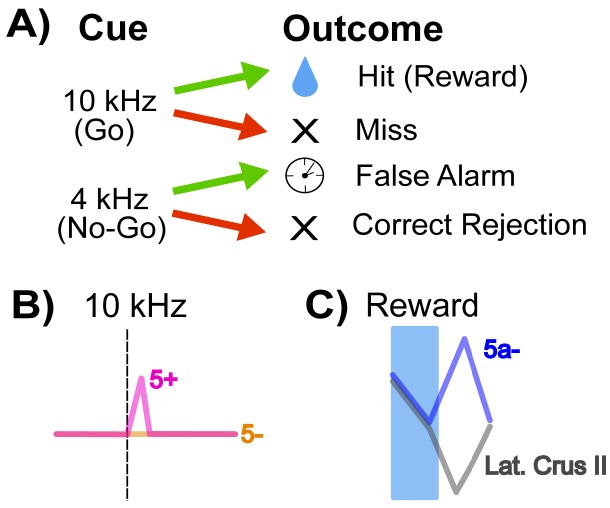
How cerebellar Crus II microzones process information during a Go/No-go task. (**A**) Schematic of the Go/No-go task. Mice are trained to associate a ‘Go’ cue – here, a 10 kHz tone – with a sweet liquid becoming available, and to react with a licking behavior. The No-go signal (a 4 kHz sound) is not associated with reward. Green arrows indicate a lick response to the signal, and red arrows that there was no lick response. A lick response to the Go tone was rewarded with food (‘hit’), and a lick response to a No-go tone (‘false alarm’) was punished with a time-out. (**B**) Relative activity of the dendrites of Purkinje cells in response to a 10 kHz Go tone. The pink trace shows the response of dendrites from an AldC+ cell (which expresses the enzyme Aldolase C), while the orange trace shows the reaction of an AldC- cell. The dendrites of the AldC+ cell are present in a microzone called 5+, and the dendrites from the AldC- cell are localized in the 5- microzone. (**C**) This graph indicates the relative activity of two groups of cells: an AldC- microzone (5a-; blue trace) in the medial Crus II, and the lateral Purkinje cell dendrites, which are similar across several microzones including 7+, 6-, 6+, 5- (gray trace). The activity of the cells is tracked during the reward delivery (blue shaded region) and post-reward intervals, where the difference in activity appears.

First, they looked at responses to the 10 kHz tone: during ‘hit’ trials, the activity of climbing fiber inputs was stronger for AldC+ Purkinje cells than for AldC- cells (Figure 1B). Second, they found that a higher percentage of AldC+ dendrites showed activity during any trial in which the mouse licked (hits and false alarms), suggesting that these cells play a larger role in motor behavior compared to climbing fiber inputs to AldC- Purkinje cells.

Tsutsumi et al. then compared the activity of climbing fiber inputs to the medial and lateral microzones of Crus II. The medial microzones showed more climbing fiber responses during ‘hit’ trials: these responses were positively correlated with lick rate, and corresponded to other aspects of motor execution. In contrast, climbing fibers in the lateral microzones revealed activity in response to all cues, even on correct rejection trials where licking was withheld, suggesting that these lateral signals are sensory responses.

Finally, Tsutsumi et al. examined how climbing fiber inputs responded to reward outcomes in different regions of Crus II. In all lateral regions (and a medial AldC+ region called 5+), the delivery of a reward was followed by a decrease in the activity of climbing fiber inputs. Meanwhile, there was an increase in the activity of climbing fiber inputs to one medial region (the AldC- region 5a-) after reward delivery ended (Figure 1C).

Overall, these studies help us to understand how relevant sensory cues, motor responses and reward outcomes are received by the various lobules and microzones of the cerebellum. They also provide insight into the functional differences between AldC+ and AldC- compartments. Armed with this knowledge, it may become possible to probe exactly how the cerebellum causally contributes to reward processing (and other higher-order cognitive functions) by stimulating specific climbing fiber inputs or Purkinje neuron subtypes during behavior. These studies are at the forefront of expanding cerebellum research beyond the study of motor control, with many new insights still to come.
